# A method for estimation of fundamental frequency for tonal sounds inspired on bird song studies

**DOI:** 10.1016/j.mex.2018.12.011

**Published:** 2019-01-04

**Authors:** Cecilia Jarne

**Affiliations:** Universidad Nacional de Quilmes, Departamento de Ciencia y Tecnología – CONICET, Buenos Aires, Argentina

**Keywords:** A maximum intensity of frequency decomposition method, Signal analysis, Fundamental frequency, Python code, Open source

## Abstract

In this work a simple implementation of fundamental frequency estimation is presented. The algorithm is based on a frequency-domain approach. It was mainly developed for tonal sounds and it was used in Canary birdsong analysis. The method was implemented but not restricted for this kind of data. It could be easily adapted for other sounds. Python libraries were used to develop a code with a simple algorithm to obtain fundamental frequency. An open source code is provided in the local university repository and Github.

• The algorithm and the implementation are very simple and cover a set of potential applications for signal analysis.
• Code implementation is written in python, very easy to use and modify.
• Present method is proposed to analyze data from sounds of *Serinus canaria*.

## Graphical abstract

**Figure:**
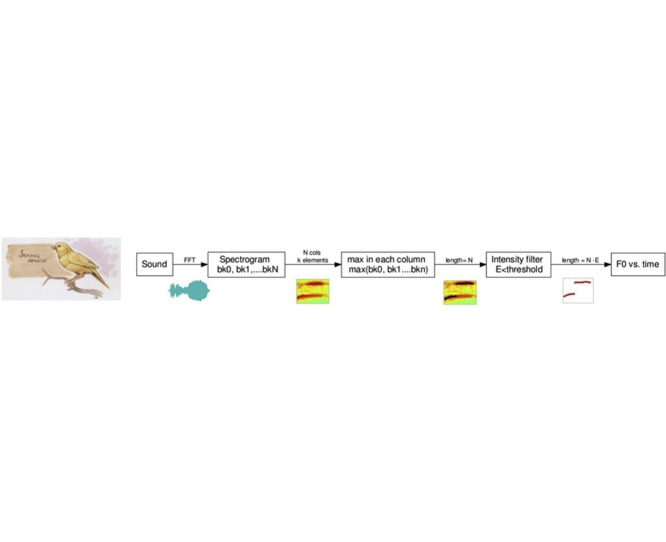

**Specifications Table**

**Table:** 

Subject area	*Select one of the following subject areas*: • *Computer Sciences*
More specific subject area	*Signal Analysis*
Method name	–
Name and reference of original method	–
Resource availability	https://github.com/katejarne/Fundamental-frequency-code http://ceciliajarne.web.unq.edu.ar/investigacion/

## Method details

### Short introduction regarding the importance of fundamental frequency of animal sounds

In the field of biology, the study of animal sounds is a key to understand behavior, evolution and the differences across the animal species. In a diversity of studies, the knowledge of fundamental frequency allows inferring information of the animal communication characteristics of the different clades.

Of particular interest is the case of bird songs. In past years birdsong has turned into a very interesting problem for the scientific community. The reason is that there are approximately 10,000 species of birds known to exist, where 4000 share with humans that the acquisition of vocalization requires a certain degree of exposure to a tutor. Hundreds of studies have focused on localizing the regions in the brain involved in the learning and production of the song. The propose is to understand through this example the mechanisms involved in the acquisition of a general and complex behavior through learning [1].

In bird songs, fundamental frequency (called f_0_) is one key on the study of peripheral mechanisms of vocalization. Besides the learning process of songbirds, there are other interesting examples where fundamental frequency is used to investigate different phenomena. For instance to analyze behavioral data. One example consists of the study of a daily oscillation in the fundamental frequency of measured on Zebra Finch song that could reveal new insights into how time of day (circadian rhythms) affects the ability to accomplish a variety of complex learned motor skills [2].

*In other species, such as sea mammals, a remarkable example is the use of funda*mental frequency in the study of acoustic signals and the supposed spoken language of the dolphins [3]. In the human case, one goal in many speech analysis applications is to follow fast variations in the fundamental frequency (f_0_) of a signal. Again, several studies were conducted in this field [[4], [5], [6], [7]].

All cases presented have in common the need to calculate accurately the fundamental frequency as an initial part of further studies. The basic problem consists of extract the fundamental frequency f_0_ from a sound signal, which is usually the lowest frequency component, or partial, that relates well to most of the other partials. In the case of a periodic waveform, most partials are harmonically related. The frequency of *this lowest partial is f_0_ of the waveform* [8].

### On the current techniques and motivation for the method

*Several implementations of fundamental frequency were applied in a variety of studies* going from music analysis to power line stability and can be found in the literature [[10], [11], [12]]. To perform this task, there are currently a lot of methods. In the case of speech, many pitch detection algorithms (PDAs) analyze a speech signal by partitioning it into segments and calculating the respective fundamental frequencies (short-term analysis) [4].

On the other hand, several methods have been proposed to obtain reliable f_0_-trajectories from harmonic signals. In this sense, fundamental frequency trackers could be classified into very general categories: autocorrelation, adaptive filters, time domain, models of the human ears and frequency domain [13].

In general, the autocorrelation algorithms consist of taking the correlation of a waveform with itself. It is expected exact similarity at a time lag of zero, with increasing dissimilarity as the time lag increases. Periodic waveforms have the following autocorrelation characteristic: the autocorrelation function is itself periodic. When the time lag increases to half of the period of the waveform, the correlation decreases to a minimum. As the time lag increases again to the length of one period, the autocorrelation again increases back to a maximum, because the waveform and its time-delayed copy are in phase. The first peak in the autocorrelation indicates the period of the waveform. These kind of methods are most appropriate at mid to low frequencies.

The difficulty with autocorrelation techniques has been that peaks occur at subharmonics as well, and it is sometimes difficult to determine which peak is the fundamental frequency and which represent harmonics or partials. The method call YIN attempts to solve these problems in several ways [14]. YIN is based on the difference function, which, while similar to autocorrelation, attempts to minimize the difference between the waveform and its delayed duplicate instead of maximizing the product (for autocorrelation).

There are also adaptive filter methods, on pitch detector for example, based on the analysis of the difference between the filter output and the filter input. This difference must be close to zero. The bandpass filter center frequency is controlled by this difference [15].

Regarding the time domain methods, one type of pitch detector is based on the analysis of the zero-crossing points. Preprocessing by filters has to be performed, in order to solve the problem of the low-amplitude zero crossings caused by high-frequency components [16,17].

With respect to pitch detectors in the frequency domain, most of them are based on the analysis of the FFT spectrum, or of the cepstrum [18]. Cepstrum analysis is a form of spectral analysis where the output is the Fourier transform of the log of the magnitude spectrum of the input waveform. The word cepstrum comes from reversing the first four letters in the word spectrum, indicating a modified spectrum. This method relies on the fact that the Fourier transform of a pitched signal usually has a number of regularly spaced peaks, that represent the harmonic spectrum of the signal. When the log magnitude of a spectrum is taken, these peaks are reduced, their amplitude cast into a usable scale, and the result is a periodic waveform in the frequency domain, the period of which (the distance between the peaks) is related to the fundamental frequency of the original signal. By applying the Fourier transform of this waveform one can get a peak at the period of the original waveform [8].

Fundamental frequency estimation is still a difficult topic in audio signal processing. There are many context-specific attempts to solve the problem, and many of them work well in their specific context. It has been difficult to develop a “context-free" f_0_ estimator. Moreover, most of the current tools are not develop in open source code. In addition, f_0_ estimators developed for a particular application, such as musical note detection or speech analysis, are well understood, but depend on the domain of the data: a detector designed for one domain is less accurate when applied to a different domain. The result is that currently there are many f_0_ estimators, few of them are appropriate to more than one domain [8] and they are not open source tools.

An example of a programming tool in open source code is called audiobio [9], but the main focus of this tool is the use in music or speech, rather than the analysis of animal sounds. Additionally, this tool is written in C, with a python interface. This means that to modify the code is necessary to program in C.

Therefore, choosing an f_0_ estimator appropriate for animal sounds or song discrimination was a difficult task. This is the reason that motivated the work. The started point was the need of a simple f_0_ tracking algorithm, easy to implement for mostly tonal sounds like the canary songs with syllables of different time scale and a frequency range between 800 Hz and 8 KHz.

This range covers the canary (*Serinus canaria*) singing range. A simple and heuristic method based on frequency-domain approaches is proposed to estimate fundamental frequency, with the main characteristic of the use of open source and optimized python libraries. The algorithm and the software implementation were designed to be used for any researcher (particularly biologist) without the need of being a signal analysis expert or deep programming knowledge.

## The proposed method

**Fig. 1:**
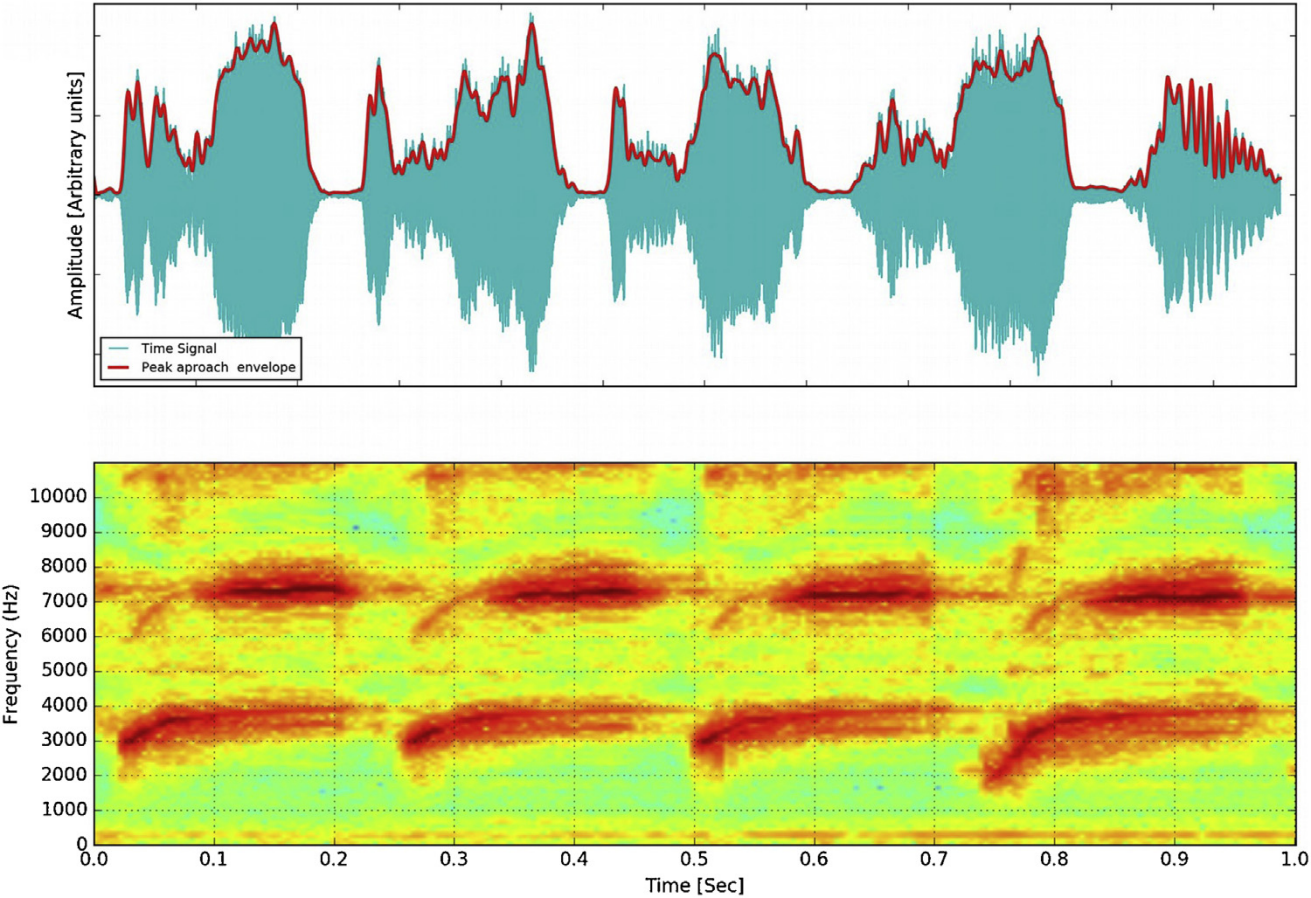
A sound Segment from Canary sound (upper panel) and its frequency content (bottom panel). Canary sound from canary examples for training [23].

The proposed algorithm is very straightforward. First, a digital audio signal is selected to perform this task. The signal spectrogram function is used over the consecutive signal segments of a preferred length to study the frequency content. A spectrogram is a visual representation of the spectrum of frequencies in a sound or other signal as they vary with time. In this representation, each point has a given value for frequency and intensity across different time bins. In general, a colored scale (or gray-scale) is used to represent the intensity of each frequency value in each time column. An example of a bird song segment and the spectrogram is shown in Fig. 1.

The main idea is to use the appropriate resolution to estimate the sonogram with a compromise between time resolution and frequency resolution. The spectrogram is used to iteratively determinate the value with the maximum amplitude for each column across each time bin (b_ki_). Then, only the value of the maximum amplitude (more intensity in the colored scale) is saved as a new variable in each temporal bin. At this point, it is possible to use the envelope audio level (E) as a threshold to discard silence parts of the signal where no sound is produced by the bird in the data. The amplitude of the signal envelope is obtained with the method described in [19]. It is necessary to use a threshold in the audio level for two reasons. First to discard the part that does not correspond to the birds singing and define the silent part. Second, because some recordings could have a noisy background, but in fact, this will depend on the quality of the recording.

The algorithm consist of the following steps:

1 To take a sound segment R(t’) and perform a sonogram with the desired resolution in time. The sonogram is called *s(t)*. Data are split into time *t’ corresponding to n* length segments and the frequency spectrum of each section is computed. Then a windowing function is applied to each segment, and the amount of overlap of each segment is specified. The sonogram is plotted as a color-map like the one shown in Fig. 1 lower panel.

(1) $$s\left(t\right)=s\left(t_{0}\right)+s\left(t_{1}\right)+s\left(t_{1}\right)+...+s\left(t_{n}\right)$$

where each s*(t_k_)* is a vector with intensity values and *m* frequency values for each t_i_ with intensity b*(f_k_)* interval:

(2) $$s\left(t_{k}\right)=b\left(f_{0}\right)+b\left(f_{1}\right)+....+b\left(f_{m}\right)$$

2 For the same sound segment, to estimate the amplitude envelope.
3 For each bin corresponding to a temporal interval *t_k_* or window, estimate the frequency where the intensity value is maximum:

(3) $$f_{fund}\left(t_{k}\right)=max_{\left(b-value\right)}\left(s\left(t_{k}\right)\right)$$

4 Save in a vector the frequencies corresponding to the maximum intensity for each temporal bin.

(4) $$f_{fund}\left(t\right)=f_{fund}\left(t_{0}\right)+f_{fund}\left(t_{1}\right)+....+f_{fund}\left(t_{n}\right)$$

5 Filter the bins with the amplitude below the threshold.

A simple schema of the algorithm is also shown in **Graphic abstract**.

Without step five, one will have the problem of keeping the maximum of all temporal bins, but in some bins there is not signal representing a sound, there is noise or silent level. In this way, to get rid of the noise and keep only the values representing the desired sound is to used a second intensity filter to consider only sounds that exceed a certain threshold.

**Fig. 2:**
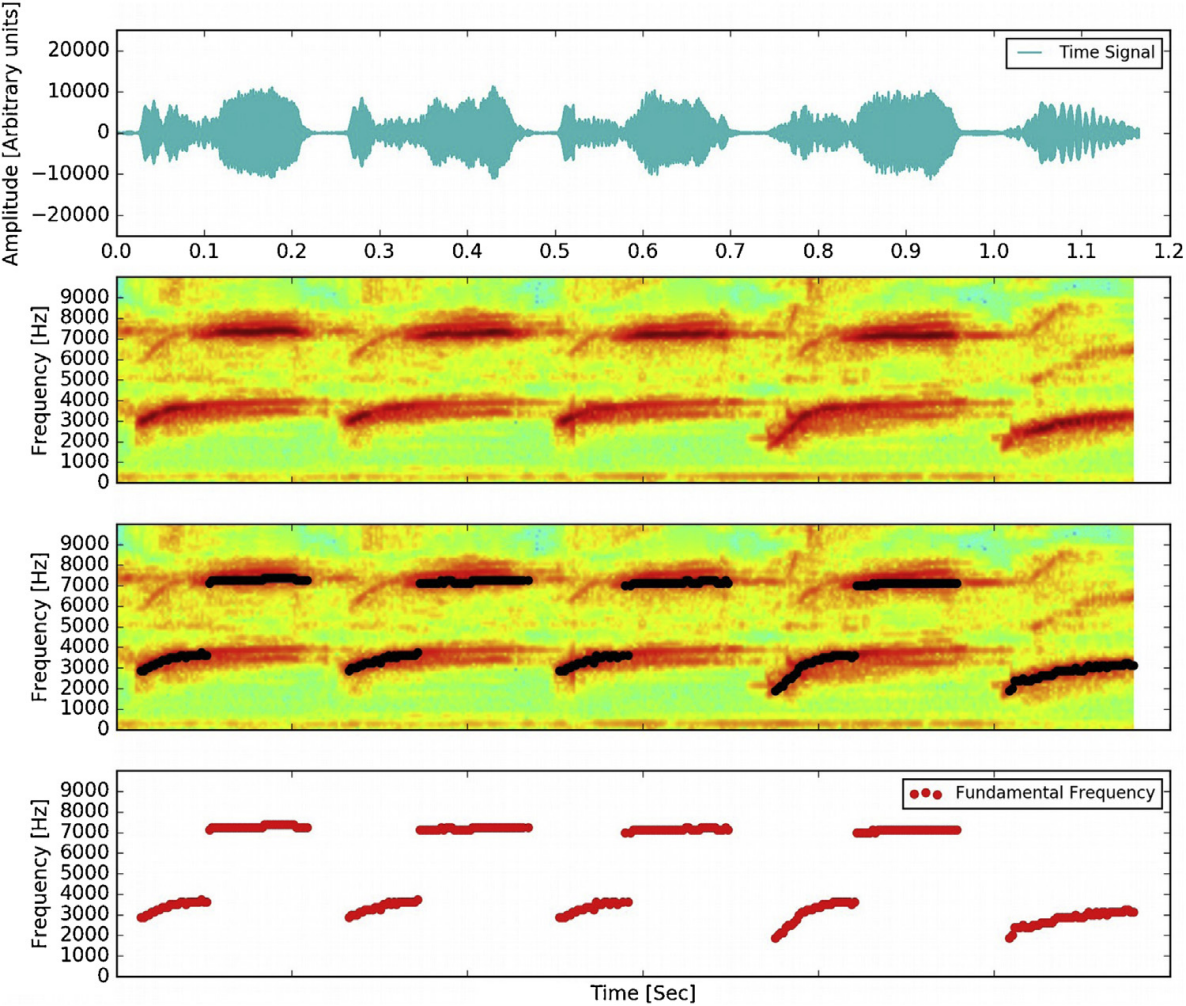
An example of the fundamental frequency estimation applied to canary sounds is shown. **First panel**: Sound amplitude of a singing segment of a *Serinus canaria* song. **Second panel**: Spectrogram. **Third panel**: Algorithm applied over the sound segment. Fourth panel: fundamental frequency vs. time.

An additional consideration is needed if we want to estimate fundamental frequency when harmonics are more intense than f_0_. This issue is not addressed here, is beyond the scope of this work. In this case, a possible solution is to use a filter over the bins to keep only frequency values between a certain range. A sound segment is used as an example to illustrate the process. The spectrogram and it is shown in the first panel of Fig. 2. In this case, it corresponds to a singing segment of a *Serinus canaria* song. It has a sample rate of 44.100 KHz and an arbitrary length of 1.15 s. The resolution of the spectrum is selected to be appropriate to the time scale of any kind of syllables of the Canary song. The syllabic rate in canaries is from 3 Hz to 30 Hz. Time resolution has to be < 1/30 = 33 ms to distinguish between different syllables, but also smaller than the duration of the shorter syllables that is 10mSec.In this way taking into account the time scale related to the sound variations, the frequency content, the frequency range, or other characteristics, the filters over intensity threshold can be used to keep the meaningful part of the signal.

## Software implementation and the application example

The software produced in this work was developed in Python. The main reason is that Python is a free widespread and open source programming language, where one can combine free and open-source math libraries such as Numpy or Scipy, two fundamental packages for scientific computing. The code developed is provided to be useful and modify by anyone. The implementation works using the function *“specgram”* in the Matplotlib libraries. It takes as argument the sound vector, the windows size, sample rate and the number of points to overlap between segments; and returns the array of amplitude, sample frequencies, and time bins via a Fast Fourier Transform. All these parameters can be tuned according to the particular application.

An appropriate amount of overlap in the sonogram part will depend on the choice of window and on your requirements. In contrast to welch's method, where the entire data stream is averaged over, one may wish to use a smaller overlap (or perhaps none at all) when computing a spectrogram, to maintain some statistical independence between individual segments [20]. After calling specgram function, a filter over the frequency bins in the sonogram is applied depending on the desired frequency range. In this stage, the Numpy function *“argmax”* is called for each temporal bin contain the intensity values and frequencies.

The value with maximum intensity is saved in a new vector. The implementation is very straightforward and the code is very short. It is provided in the University local repository with password "fundamental". It is possible to use the Python 2 or modify the code for Python 3. Also, an output of the signal plots in the required timescale is provided in. jpg format together with a table output of the frequency vs time values in. txt format.

**Fig. 3:**
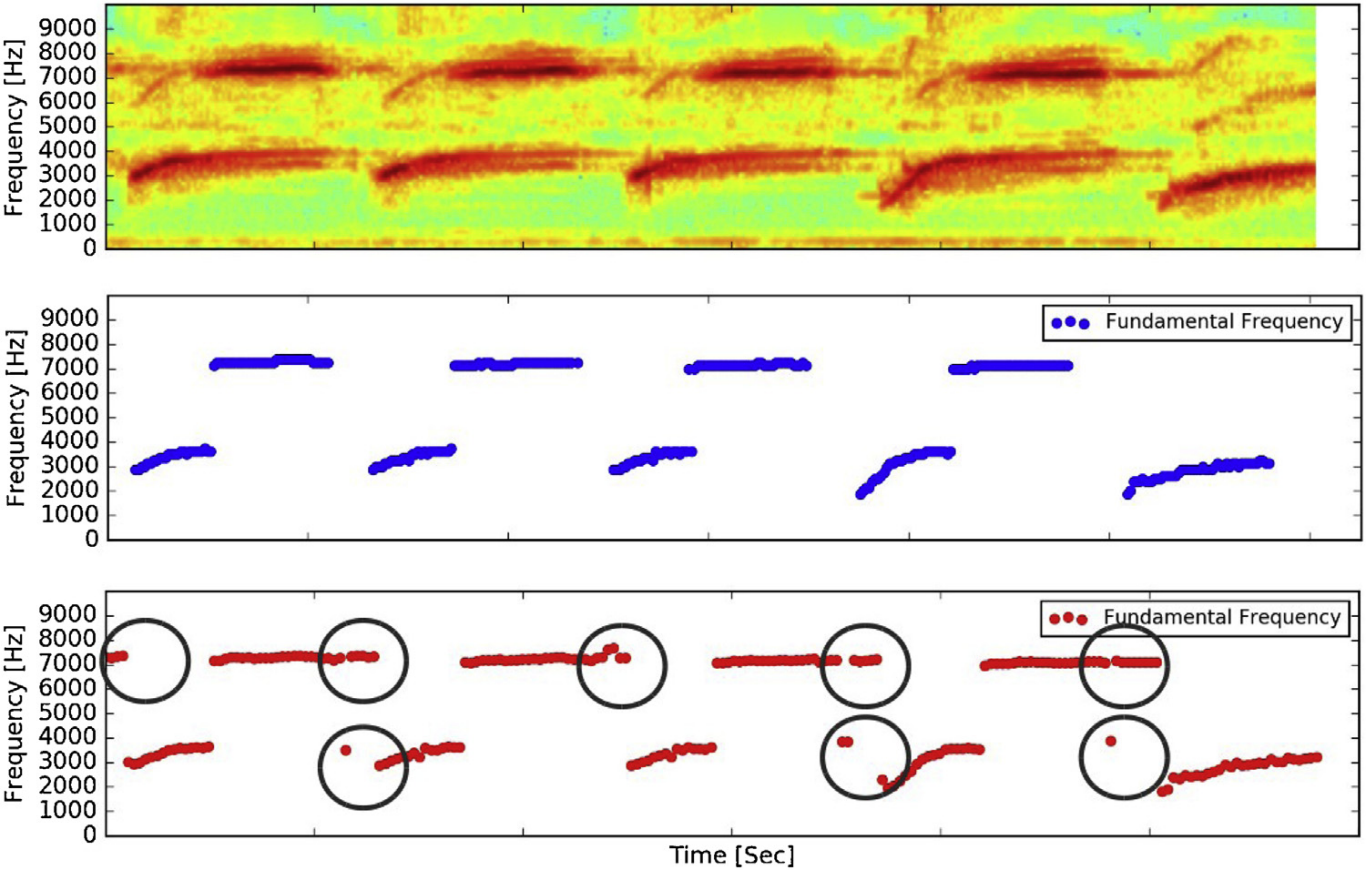
Audio segment corresponding to *Serinus canaria*. **First panel**: Song spectrogram. **Second panel**: Algorithm develop in this work applied over the sound segment. **Third panel**: Naive implementation to obtain fundamental frequency vs. time from reference. The bubbles show the problem of not be able to filter noise.

The algorithm is very fast the implementation easy. For instance, it took 12.3 seconds to obtain fundamental frequency in a signal of 20 s duration, including the creation of the corresponding plots and. txt file. The code only uses the standard libraries Scypi, Numpy and Matplotlib. The algorithm was compared the a similar algorithm, with also a simple Python implementation with a version of the code that was found in [21]. Unfortunatly the implementation lacks of proper documentation and also has no support for stereo files. Also, no treatment for noise is performed. It used also and additional library called Pyaudio [22] to perform the task. Results obtained with both are similar but it has no clear mechanism to filter properly silence parts of the recording. They are shown in Fig. 3. In this way, here is presented a simple and tunable solution to be applied for different data analysis.

## Additional information

This work was motivated by the search for alternatives to obtain fundamental frequency with a simple method. To perform that task, a review of current methods was performed.

Another main motivation of present work is to introduce an algorithm that was implemented in the open source code and pure python. The idea was to use python code to make it more easy to modify, given that the community in biology is more proficient in that programming language.

For tonal birdsong, the algorithm performs well. It can be used for other animal sounds or another kind of signals, but it has the limitation of the richness of the spectrum or the noise.

Special consideration is the case when the fundamental frequency is different from the maximum intensity value. For initial analysis, this code could work as a starting point that can be combined with other strategies to isolate and track the variations of the desired f_0_, but is not the scope of present work.

The principal advantage of this software is that documentation of the code is provided as with clear statement of how each part works. Also, data analysis is moving in the open source and collaborative software developments. Further work could include methods to isolate fundamental frequency for more complex cases.

## Funding

This work was developed without additional funding other than the Posdoc Salary granted for CONICET and Universidad Nacional de Quilmes.

## Conflict of interest

The authors declare that there are no conflicts of interest.

## Supplementary material and/or additional information

Available on the repository.

## References

[1] Mindlin Gabriel B., Laje Rodrigo (2005). The Physics of Birdsong.

[2] Wood W.E., Osseward P.J., Roseberry T.K., Perkel D.J. (2013). A daily oscillation in the fundamental frequency and amplitude of harmonic syllables of Zebra finch song. PLoS One.

[3] Mallawaarachchi Asitha, Onga S.H., Chitre Mandar, Taylor Elizabeth (2008). Spectrogram denoising and automated extraction of the fundamental frequency variation of dolphin whistles. J. Acoust. Soc. Am..

[4] Staudacher Michael, Steixner Viktor, Griessner Andreas, Zierhofer Clemens (2016). Fast fundamental frequency determination via adaptive autocorrelation. J. Audio Speech Music Proc..

[5] Rashidul Hasan M.A.F.M., Shimamura Tetsuya (2018). A fundamental frequency extraction method based on windowless and normalized autocorrelation functions. Conference: Proc. WSEAS Int. Conf. Circuits, Systems, Signal and Telecommunications.

[6] Roa Sergio, Bennewitz Maren, Behnke Sven (2007). Fundamental frequency estimation based on pitch-scaled harmonic filtering. IEEE International Conference on Acoustics, Speech and Signal Processing.

[7] Joseph Timoney, Thomas Lysaght (2001).

[8] David Gerhard. Technical Report TR-CS 2003-06, ISSN 0828-3494. Pitch Extraction and Fundamental Frequency: History and Current Techniques http://www2:cs:uregina:ca/gerhard/publications/TRdbg?Pitch.pdf.

[9] Audibio software. Https://aubio:org/documentation.

[10] Mishra Shubham, Yadav Rahul, Mukesh Nitin (2016). Pitch extraction and fundamental Frequency estimation from indian Folk music using time domain and Frequency domain technique. Int. J. Latest Trends Eng. Technol..

[11] Huang Jingchang, Zhang Xin, Zhou Qianwei, Song Enliang, Baoqing Li. (2014). A practical fundamental frequency extraction algorithm for motion parameters estimation of moving targets. IEEE Trans. Instrum. Meas..

[12] Suresh Babu P., Jayaram Kumar S.V. (2009). A novel algorithm to extract exact fundamental frequency components during faults for digital protection of power system. ARPN J. Eng. Appl. Sci..

[13] Rossignol Stephane, Desain Peter, Honing Henkjan (2001). State-of-the-art in fundamental frequency tracking. Proceedings of the Workshop on Current Research Directions in Computer Music.

[14] de Cheveigne Alain, Kawahara Hideki (2002). Yin, a fundamental frequency estimator for speech and music. J. Acoust. Soc. Am..

[15] Lane J. (1990). Pitch detection using a tunable IIR lter. Comput. Music. J..

[16] Moorer J.A. (1975). On the Segmentation and Analysis of Continuous Musical Sound, Ph-D Thesis.

[17] Hermes D., Cooke M., Beet S. (1992). Pitch analysis. Visual Representation of Speech Signals.

[18] Schafer R., Rabiner L. (1970). System for automatic formant analysis of voiced speech. J. Acoust. Soc. Am..

[19] Jarne C. (2017).

[20] Scipy Spectrogram Library documentation: https://docs.scipy.org/doc/scipy-0.16.0/reference/generated/scipy.signal.spectrogram.html.

[21] Code implementation in: https://stackoverflow:com/questions/2648151/python?-frequency-detection.

[22] Audio Analysis libraries http://people.csail.mit.edu/hubert/pyaudio/.

[23] Canary sound from canary examples for training: https://www:youtube:com/watch?v=qnZ-gj7Si2w&t=21s.

